# HIV Treatment as Prevention: Natural Experiments Highlight Limits of Antiretroviral Treatment as HIV Prevention

**DOI:** 10.1371/journal.pmed.1001231

**Published:** 2012-07-10

**Authors:** David P. Wilson

**Affiliations:** The Kirby Institute, Faculty of Medicine, University of New South Wales, Sydney, New South Wales, Australia; Duke University Medical Center, United States of America

## Abstract

There is growing enthusiasm for increasing coverage of antiretroviral treatment among HIV-infected people for the purposes of preventing ongoing transmission. Treatment as prevention will face a number of barriers when implemented in real world populations, which will likely lead to the effectiveness of this strategy being lower than proposed by optimistic modelling scenarios or ideal clinical trial settings. Some settings, as part of their prevention and treatment strategies, have already attained rates of HIV testing and use of antiretroviral therapy—with high levels of viral suppression—that many countries would aspire to as targets for a treatment-as-prevention strategy. This review examines a number of these “natural experiments”, namely, British Columbia, San Francisco, France, and Australia, to provide commentary on whether treatment as prevention has worked in real world populations. This review suggests that the population-level impact of this strategy is likely to be considerably less than as inferred from ideal conditions.

## Introduction

HIV prevention decision-makers across the world are considering the expansion of antiretroviral therapy (ART) for HIV-infected people in order to reduce their infectiousness and thus prevent onward transmission. This approach, called treatment as prevention, is a paradigm shift from using ART for the sole purpose of improving the health and longevity of patients with HIV. We are now in an era where the secondary benefit of ART is being considered as potentially the primary public health approach to controlling HIV epidemics.

Several findings suggest that treatment might be effective as prevention: the HPTN 052 study demonstrated that ART reduces sexual transmission between discordant couples in a trial setting [1]; various ecological studies from community settings have shown an association between ART programs and reduced markers of incidence [2]–[5]; associations have been demonstrated between reduced viral load and lower infectiousness [6]–[8]; and some theoretical models even suggest that under idealised conditions, elimination might be possible [9],[10]. However, these findings do not imply that widespread scale-up of ART programs under real world conditions will reduce HIV incidence at a population level to the degree that some people are expecting (i.e., towards elimination). Cluster-randomised trials are currently underway in Africa to investigate the impact of high coverage of ART at the population level. In the meantime, models are projecting potential epidemic trajectories associated with treatment-as-prevention strategies under less ideal conditions [11], and various national and international organisations are already discussing operational issues about how to implement treatment as prevention [12].

We do not need to wait for trials of increased ART coverage to be completed, or speculate through the use of mathematical models, to have some understanding of the likely population-level impact of this strategy. Treatment as prevention has essentially been implemented in some settings already for a considerable time. Planned treatment-as-prevention approaches involve frequent universal testing and initiation of ART early post-diagnosis, but increasing treatment coverage at any stage of infection—and reaching high degrees of viral suppression across a population of people living with HIV—is de facto treatment as prevention. Some settings have achieved these objectives as part of their independent prevention and treatment responses: these settings can be considered as natural experiments for treatment as prevention at the population level.

## Natural Experiment Case Studies

### British Columbia, Canada

A study by Montaner et al. [3] has been widely promoted as demonstrating treatment as prevention in a community setting, namely, among people who inject drugs (PWID) in British Columbia, Canada. In British Columbia, there is universal access to free rapid HIV testing (though it is not known what proportion of PWID get tested for HIV each year). Guidelines for ART in British Columbia indicate that any HIV-positive patient may commence treatment, regardless of CD4 count, and ART is recommended for all symptomatic patients with established disease, and for asymptomatic individuals with CD4 cell count ≤500 cells/µl [13]. Estimates for ART coverage are difficult to quantify precisely, but coverage is considered to be relatively high and has certainly increased over time.

Montaner et al. found that there was an association between declining rates of HIV diagnoses and increasing rates of testing, ART coverage, and viral suppression. However, it is not clear to what extent the reduction in incidence is attributable to ART versus other interventions. As discussed by Smith et al. [14], also in the July 2012 *PLoS Medicine* Collection, “Investigating the Impact of Treatment on New HIV Infections”, analyses conducted for British Columbia have been ecological, and declines in incidence could be attributed to other prevention programs specifically targeting this population group over the same period [15].

### San Francisco, United States

With high, and increasing, rates of HIV testing and ART coverage and effectiveness, San Francisco is an obvious case study for evaluating the role of treatment as prevention. It is estimated that rates of HIV testing have been increasing in San Francisco, such that ∼72% of the core group at risk of infection, namely, men who have sex with men (MSM), received an HIV test in the past 12 months, and only 15%–20% of HIV cases are undiagnosed [4]. An increasing proportion of HIV-infected patients are enrolled in care (∼71% in 2004 and 78% in 2008) [16], and mean levels of community viral load have significantly decreased (from ∼25,000 copies/ml in 2004/2005 to 15,000 copies/ml in 2008) [4].

Das et al. [4] used San Francisco's HIV/AIDS surveillance system to examine trends in community viral load and new HIV diagnoses, as a surrogate marker for incidence. They found that reductions in community viral load were associated with decreased HIV diagnoses since 2004. As a purely ecological study, causation cannot be attributed to ART, but their results suggest that high coverage of ART could have reduced HIV transmission at the population level. However, although the number of newly diagnosed and reported HIV cases has been declining in San Francisco, the rate of new infections is still relatively high [4], possibly because of the substantial HIV prevalence (∼25%) among MSM [17],[18]. As such, even if the average per individual infectiousness is reduced, there is still likely to be a significant number of new HIV infections occurring at the population level each year.

### France

A “treatment as prevention” statement has been released by the French National AIDS Council [19], which takes a less assertive approach to “test and treat” but still strongly promotes testing and treatment. The level of undiagnosed infections in France is approximately 25%–30%, comparable to levels in other resource-rich settings [20]–[28]. ART guidelines in France in 2007 indicated that ART should be started as early as possible for symptomatic patients and those with high viral loads (>100,000 copies/ml), and for asymptomatic patients when the CD4 count reached 350 cells/µl [29]. There have been significant increases in the uptake of ART among eligible people in France (to ∼85%), and ∼92% of treated patients achieve plasma viral suppression [30]. However, treatment-as-prevention strategies cannot be said to have been fully implemented in France, as many patients initiate ART too late [29].

The outcomes of the natural experiment in France suggest that there may be differences between at-risk groups in the population-level effectiveness of ART for reducing incidence: HIV incidence has declined or remained stable in all major population groups, except MSM, where incidence has been high and increasing [30]. Data from behavioural studies indicate that unprotected anal sex and numbers of sexual partners among MSM have increased [31] (also coinciding with increases in syphilis transmission [32]), raising the possibility that disinhibition or independent sociobehavioural changes could undercut the effectiveness of treatment as prevention. It is also possible that the increased HIV incidence among MSM could be due to higher risk behaviours among those who are not on ART and do not have suppressed virus.

### Australia

Australia could also be considered a setting where a natural experiment for treatment as prevention has taken place. First, the HIV epidemic is highly concentrated, with the majority (∼80%) of all HIV cases being among MSM [33], a population generally well educated and actively engaged with respect to HIV. Second, HIV testing is routinely carried out by most MSM, with approximately 60%–75% of men self-reporting an annual test [34] and just 10% of men reporting having never been tested [35]. Third, all regimen lines and combinations of ART are publicly funded and freely available to all HIV-infected patients. Australian guidelines for treatment advise considering ART when CD4 cell count is <500 cells/µl, and definitely treating when CD4 cell count is <350 cells/µl. There are increased numbers of people receiving ART in Australia (at about 70% of all individuals living with HIV) [36]; however, about 20% of individuals commence ART when CD4 cell count is <200 cells/µl, because of late presentation [33]. Fourth, the proportion of people on ART with undetectable viral load has increased from 65% to 90% (at 400 copies/ml sensitivity; 40% to 85% at 50 copies/ml sensitivity) [37]. Further information and analyses on these data are provided elsewhere [38].

It is likely that many countries would aspire to the conditions in Australia as a target for treatment as prevention, as this is a real world population with high coverage of effective treatment. However, new HIV diagnoses, which can be interpreted as reflecting HIV incidence [39], have increased from around 700 cases in 1999 (a nadir of national diagnoses) to around 1,000 new cases annually [33]. This suggests that implementation of treatment as prevention may have less impact on reducing population incidence than previously expected.

## Limits to Treatment as Prevention

Treatment as prevention possibly has the greatest chance to succeed now in resource-rich countries with concentrated HIV epidemics, where there is generally universal access to ART, adequate infrastructure, and guidelines that enable early initiation of treatment. However, it is in these very settings that HIV incidence, or surrogate markers thereof, has been increasing [40]–[45], as in Australia and France. Indeed, at the latest Annecy Group meeting (consisting of representatives from national HIV/AIDS surveillance organisations from developed countries in North America, Australia, Western Europe, and the UK) in Rome in January 2011, it was ascertained that despite differences in epidemiological profiles, surveillance systems, and programmatic responses, HIV epidemics among MSM were generally stable or increasing in almost all of these developed-country settings, despite widespread and increasing availability and effectiveness of ART. Outbreaks of HIV among PWID have also recently been observed in some of these countries [46],[47].

There are numerous possible reasons for the apparent ineffectiveness of increased treatment in reducing HIV incidence in “natural experiments”. One potential explanation is changes in risk behaviour (shifts in cultural practices, condom fatigue, or risk compensation), as observed in numerous settings including France and Australia [48]. Another possible explanation is the influence of migration from higher prevalence regions, which leads to greater numbers of detected cases in the country of question—sometimes used as a measure of incidence—as well as greater background prevalence. There is also the potential emergence of marginalised groups that experience additional barriers to accessing services. These marginalised groups often include migrant and other populations that experience relatively high levels of stigma and discrimination but that are also at greatest risk of HIV infection. Increases in prevalence of other sexually transmitted diseases can also increase HIV incidence, since some sexually transmitted diseases act as a biological cofactor for increasing both HIV infectiousness and susceptibility [49],[50]. Another possibility is that treatment is not as effective in reducing infectiousness for riskier modes of transmission as it is for heterosexual transmission (the only mode of transmission considered in the HPTN 052 study) [4],[51]–[53]. Currently, there is little evidence that treatment as prevention is as effective for MSM and PWID [54],[55]. These factors may help explain the observed increases in HIV incidence in the era of expanded ART.

**Figure 1:**
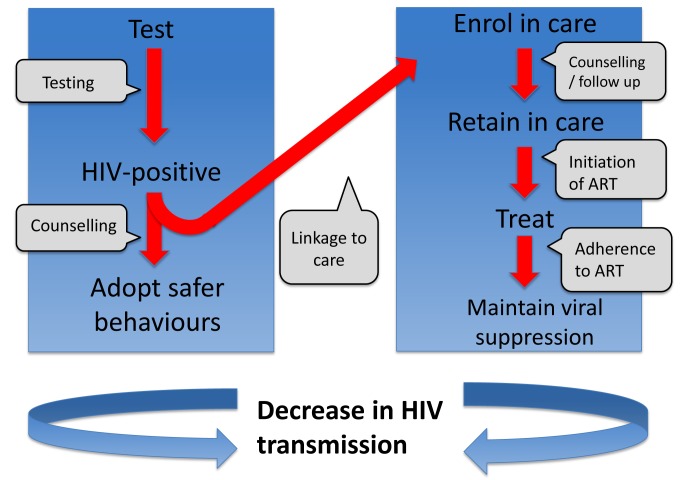
Series of steps required in order to reduce onward transmission from someone infected with HIV.

One way to consider the problem is that there is a series of barriers to overcome for treatment to be effective in reducing infectiousness (Figure 1). As indicated by Gardner et al. [56], treatment can have a population-level effect in prevention if a high proportion of all HIV-infected people (i) are tested for HIV, (ii) are linked to clinical care in a timely manner, (iii) are retained in care, (iv) receive effective ART, and (v) are adherent to treatment and regularly monitored. It is not uncommon for people to drop out at any of these barriers. Idealised conditions for a treatment-as-prevention strategy may involve setting targets of 90% of all people at each barrier progressing to the next stage. However, as pointed out by Gardner et al., this would result in a maximum of just 66% of HIV-infected people in the population having suppressed virus. Populations of people on ART may have reduced transmission potential, but transmission events are still likely to occur from individuals on ART, as well as from the remaining HIV-infected population without suppressed virus [57].

**Figure 2:**
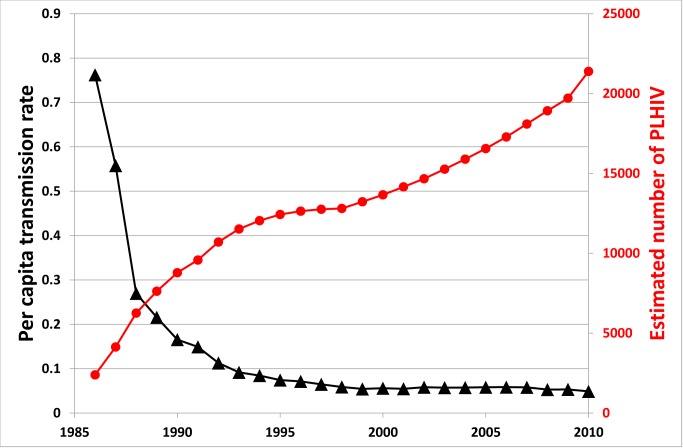
Estimated number of people living with HIV in Australia and per capita transmission rate over time. The per capita transmission rate is defined as the average number of new onward HIV infections resulting from each HIV-infected person per year; this is calculated as the number of new diagnoses in a given year (as a surrogate marker for incidence) divided by the estimated number of people living with HIV (PLHIV).

A related problem of treatment as prevention is that the significant advances in the effectiveness of ART in reducing viral replication have decreased HIV/AIDS-associated mortality [58],[59], thereby resulting in a growing pool of HIV-infected people. There is a balance between ART reducing infectiousness and increasing prevalence. This is demonstrated in the natural experiment case of Australia. As shown in Figure 2, the estimated average number of onward HIV infections resulting from each HIV-infected person per year has decreased substantially, but has levelled off at a value above zero. At the same time, the prevalence of HIV has been steadily increasing in Australia because of increased survival due to effective ART (the trend is not altered considerably when adjusted for population size). Correspondingly, overall population incidence has increased over this period. Also, acute HIV infection, with high viremia and high infectiousness, is likely to be an important contributing factor to ongoing transmission [60]–[63], particularly as most of these cases are usually unrecognised.

On the positive side, the potential problem of there being an increased pool of potential transmitters produced by successful ART may turn out to be minor. People's sexual-transmission-related behaviours generally decrease as they age. Therefore, the average transmission rate per infected person may not be reflective of transmission from the majority of people living with HIV. It may be possible to reduce the average reproduction number, *R*, to below the elimination threshold, *R*<1, even with higher prevalence. Future studies may be able to assess whether this is feasible under realistic conditions.

It is important to note that over the last 5–10 years there have been substantial increases in ART programs across low- and middle-income countries. There is now clear evidence of decreasing HIV prevalence across eastern and southern Africa, which is undoubtedly multifactorial but may reflect some impact of ART on transmission (as assessed by Johnson et al. [64] for South Africa). There have also been large reductions in mortality and corresponding reductions in prevalence throughout Asia associated with ART programs (e.g., [65]–[67]). Although such benefits are to be celebrated, and there is little doubt that ART programs have likely had an impact in reducing incidence, the levels of undiagnosed infections and treatment coverage make it unlikely that treatment as prevention can lead towards elimination at this stage.

## Conclusions

The efficacy of treatment in reducing transmission has been demonstrated for heterosexual transmission in the HPTN 052 trial, with supporting evidence from other types of studies. However, this does not imply that increased ART coverage will result in substantial declines in incidence in real world populations. The average per person rate of transmission will decrease because of ART, but it will likely saturate at a level above zero. Due to increased prevalence of potential transmitters, and other limitations, it may be difficult to decrease overall population incidence without other prevention approaches. While trial results are obtained under optimised conditions, where regular counselling and condoms are provided and where there are relatively low rates of sexually transmitted infections, this is often not the situation in the real world. There are also other external factors that may limit the impact of treatment as prevention, including adherence to treatment and shifts in sexual behaviours.

Justifiably, there is large enthusiasm for treatment as prevention. But current planning is based on expected outcomes informed by clinical trials and models—with supporting evidence from ecological and observational studies—that may be overly optimistic. Natural experiments suggest that there are limitations to the overall benefit that can be achieved with this strategy. Before large portions of HIV/AIDS budgets [68] are shifted to treatment as prevention in place of traditional prevention approaches, these limitations need to be given appropriate consideration. It must be acknowledged that ART is cost-effective with respect to clinical benefits [69],[70] and is likely to be even more so if prevention benefits are included. But combination prevention using other approaches proven to be effective, feasible, and cost-effective is also essential to reduce incidence among all major groups at risk of infection.

**Key Points**

- The real world effectiveness of treatment as prevention is likely to be less than the efficacy measured in trials or calculated in optimistic model scenarios.
- Some settings have attained rates of testing and effective ART coverage that many countries would aspire to as targets for treatment-as-prevention strategies.
- Examination of data from treatment-as-prevention “natural experiments” suggests that there are limitations to reductions in population incidence.
- Limitations might stem from behaviour changes, difficulties linking patients with and retaining them in clinical care, differences in the effectiveness of ART for different modes of HIV transmission, and the increasing pool of potential transmitters produced by successful ART.

## Abbreviations

ART: antiretroviral therapy
MSM: men who have sex with men
PWID: people who inject drugs
